# Reply to: Considerations of emerging diaphragmatic ultrasound techniques for clinical practice: potential pitfalls and future challenges

**DOI:** 10.1183/16000617.0064-2026

**Published:** 2026-04-29

**Authors:** Ivo Neto Silva, Karim Bendjelid

**Affiliations:** 1 Intensive Care Division, Department of Acute Medicine, Geneva University Hospitals, Geneva, Switzerland; 2 Geneva Hemodynamic Research Group, Faculty of Medicine, University of Geneva, Geneva, Switzerland; 3 Care Directorate, Geneva University Hospitals, Geneva, Switzerland; 4 Centre of Physical Activity, Health and Leisure (CIAFEL), Faculty of Sport, University of Porto, Porto, Portugal

## Abstract

We thank H. Zhao and co-workers for their thoughtful and constructive comments on our recent integrative review [1], which aimed to synthesise emerging diaphragm ultrasound techniques across clinical contexts with an emphasis on reliability, physiological interpretation and clinical applicability. We welcome the opportunity to further clarify several methodological and physiological aspects raised in their correspondence.

## Shareable abstract

**Emerging diaphragm ultrasound techniques provide complementary insights into respiratory muscle function. Careful physiological interpretation and methodological standardisation remain essential for meaningful use of PW-TDI and shear wave elastography.** https://bit.ly/4baEqE0

*Reply to H. Zhao and co-workers:*

We thank H. Zhao and co-workers for their thoughtful and constructive comments on our recent integrative review [1], which aimed to synthesise emerging diaphragm ultrasound techniques across clinical contexts with an emphasis on reliability, physiological interpretation and clinical applicability. We welcome the opportunity to further clarify several methodological and physiological aspects raised in their correspondence.

An important clarification relates to comparisons between M-mode-derived metrics and pulsed-wave tissue Doppler imaging (PW-TDI) parameters. Although both assess diaphragm motion, they rely on different measurement constructs: M-mode excursion reflects global diaphragmatic displacement over time, whereas PW-TDI measures instantaneous local tissue velocity within a specific sampling volume. Consequently, direct interchangeability between parameters derived from these techniques should not be assumed, as their relationship is unlikely to be linear. For example, velocity–time integral (VTI) from PW-TDI provides a displacement-equivalent parameter, yet in a weaning study it did not consistently approximate M-mode excursion [2]. Accordingly, PW-TDI may be viewed as a granularity-enriched approach providing enhanced insight into diaphragmatic displacement kinetics [3], complementing conventional excursion-based assessment, as highlighted by H. Zhao and co-workers.

Furthermore, PW-TDI measurements are strongly influenced by probe orientation, fibre alignment and tissue sampling, such that recorded motion reflects only fibres within the Doppler region. During assisted ventilation, displacement may result from both active contraction and passive ventilator-driven movement, and variability in ventilatory support may affect velocity measurements and reproducibility.

Peak diaphragm contraction velocity (peak-DCV) correlates with parameters of transdiaphragmatic pressure and may reflect inspiratory force generation; however, motion-derived ultrasound indices integrate neural respiratory drive, loading conditions and thoracoabdominal mechanics rather than isolated contractile properties. Increases in peak-DCV reported in patients with weaning failure [2, 4] should therefore be interpreted cautiously. Consistent with this complexity, diaphragmatic excursion has shown limited association with respiratory effort in both assisted and unassisted breathing conditions [5, 6].

For these reasons, we do not consider the reported divergence between higher peak-DCV values and lower mean diaphragm contraction velocity (mean-DCV, from M-mode) to represent a physiological contradiction. Rather, excursion-over-time metrics are influenced by respiratory drive and mechanical conditions, and therefore should not be interpreted as direct indicators of diaphragmatic endurance as mentioned. In evolving respiratory muscle failure, compensatory strategies may favour increased respiratory frequency rather than greater displacement, resulting in rapid shallow breathing patterns reflected in indices such as the rapid shallow breathing index [7]. Increases in peak-DCV may thus represent adaptive breathing responses to increased load; interpretation should therefore remain grounded in the broader physiological context and consider ultrasound acquisition conditions.

Shear wave elastography (SWE) bridges imaging and biomechanics, and offers potential for advanced assessment of respiratory muscle mechanical properties. As noted in the correspondence, muscle anisotropy is a key factor influencing measurements [8, 9].

While anisotropy, reflecting the directional dependence of mechanical behaviour relative to muscle fibre orientation, is an inherent biomechanical feature of skeletal muscle, the need for multidirectional SWE assessment should be balanced against clinical feasibility, reproducibility and incremental diagnostic value. The diaphragm's unique geometry and architecture introduce additional challenges for consistent multidirectional measurements compared with the limb muscles commonly studied with elastography. We agree that dual-direction SWE assessment may enrich biomechanical characterisation. Nevertheless, in clinical practice, a technically robust and reproducible single-plane approach may remain clinically informative and should not be considered insufficient, particularly when supported by reproducibility data. Further studies are needed to clarify the clinical implications of multidirectional measurements.

Acquisition in a plane transverse to the muscle fibres may pose technical challenges related to diaphragmatic motion and intercostal acoustic windows. Depending on probe positioning, breathing pattern and underlying respiratory conditions (*e.g.* hyperinflation or diaphragm flattening), consistent visualisation may be difficult to maintain. Transverse measurements have shown lower inter-operator reproducibility than longitudinal acquisitions, whereas longitudinal orientation has demonstrated good to excellent reliability in both healthy individuals and critically ill patients [10].

Regarding probe orientation, our interpretation of cited studies differs from that proposed in the correspondence. Aarab *et al*. [11] explicitly describe rotation of the probe to achieve alignment parallel to diaphragm muscle fascicles, consistent with prior reliability studies from the same group [10]. More broadly, diaphragmatic SWE measurements depend strongly on acquisition conditions, including probe orientation/positioning and respiratory phase, as stiffness reflects both passive tissue properties and active muscle state; these factors should be explicitly considered when interpreting SWE-derived stiffness values.

Emerging diaphragm ultrasound techniques provide a multidimensional perspective integrating structural, mechanical and functional domains. Clinical translation requires methodological standardisation, clearer physiological interpretation and validation of meaningful outcomes. Until stronger evidence clarifies relationships between modalities, cautious interpretation remains essential.
